# Grand Challenges in Radiology

**DOI:** 10.3389/fradi.2021.615138

**Published:** 2021-04-20

**Authors:** Dinggang Shen

**Affiliations:** School of Biomedical Engineering, ShanghaiTech University, Shanghai, China

**Keywords:** artificial intelligence, cardiothoracic imaging, interventional radiology, diagnostic radiology, emergency radiology

Radiology has been significantly reshaped and innovated by advancements of technologies, such as imaging instruments, artificial intelligence (AI), and information technology, and it has also been substantially impacted by its interactions with other disciplines. Therefore, our perspective of grand challenges in radiology is mainly concerned with technological and multidisciplinary obstacles to overcome in the years to come. We will address specific challenges associated the six sub-fields of radiology, namely, neuroradiology, diagnostic radiology, interventional radiology, emergency radiology, cardiothoracic imaging, and AI in radiology.

## Grand Challenges in Neuroradiology

Modern neuroimaging technologies and protocols generate much information such as various types of parametric maps and activity patterns on the human brain (1). One of the grand challenges is how to best represent and visualize such rich information to neuroradiologists so that they will take the full advantage of available information and do not feel overloaded. Developing novel brain mapping methods and tools will be very helpful to represent and visualize such multimodal parametric maps and activity information in a more neuroanatomically meaningful context (2), which will fundamentally assist neuroradiologist to understand and interpret. Neuroradiologists frequently interact with other disciplines such as neurology, neuropathology, neurosurgery, neuro-oncology, and psychiatry, and thus developing new paradigms and tools that can enable, facilitate, and support such multidisciplinary interactions and discussions will be enormously useful. Neuroradiology is naturally rooted in neuroscience, and brain science information sources from molecular-level genes/genomes to human behaviors could be relevant to neuroradiology. It would be a daunting yet important task for neuroradiologists to integrate and comprehend such complex brain science information into their practice. In this sense, efficient integration and representation of brain science knowledge and discoveries into neuroradiology is a grand challenge.

## Grand Challenges in Diagnostic Radiology

Diagnosis radiology provides a main source of information for assessment of human diseases and it plays a major role in clinical diagnosis and disease management. Advancements in biomedical engineering and biotechnology in the past few decades have offered novel ways of acquiring in-depth information about human diseases such as molecular imaging and genomics. How to fuse and integrate such multiscale and multimodal information into diagnostic radiology is a grand challenge. Also, diagnostic radiology has been frequently interacting with other disciplines such as nuclear medicine, molecular imaging, neurology, oncology, cardiology, pathology, surgery, and laboratory medicine, and it will be vitally important to develop new approaches and paradigms for diagnostic radiologists to collaborate with other physicians as a team. Finally, in the era of big data and AI, how to improve the accuracy, efficiency and productivity in diagnostic radiology by incorporating novel information technology and AI systems will be a grand challenge, which will be discussed from an AI angle in the last section.

## Grand Challenges in Interventional Radiology

Interventional radiology is associated with a wide range of biomedical imaging modalities (such as X-rays, MRI, fluoroscopy, CT, and ultrasounds) and interventional procedures (such as treating tumors, biopsies, and placing stents) to make diagnosis and treatment inside of the human body. Thus, one of the grand challenges in interventional radiology is integration and fusion of imaging multimodalities within the context of interested tissues or organs, in nature. Thus, developing smart and informative interventional navigation systems will be a key to meet such needs. Second, interventional radiology is at the frontier of integrated diagnosis and therapy, thus integration of novel technologies in targeted drug delivery, molecular targeted imaging and therapeutics, and AI-enable target recognition into interventional procedures will be quite challenging yet rewarding. Third, in interventional radiology, a big challenge and also a big next step is how to improve accuracy of targeted interventional procedures through the introduction and integration of imaging-compatible medical robots (3). Such robots-assisted interventional radiology will bring in a variety of benefits for patients and physicians.

## Grand Challenges in Emergency Radiology

Emergency radiology is defined and characterized by rapid imaging scan and quick turnaround of radiological reading (4). Main technical challenges are associated with these two defining characteristics. First, developing rapid imaging scan setup and fast data acquisition technologies will be of main interest and concern in emergency radiology, which will significantly increase imaging capacity and reduce risk of delaying emergent patient management. Second, developing novel AI technologies and systems can significantly reduce emergency radiologists' burden and improve their scan reading efficiency. Such user-friendly AI systems can substantially benefit emergency radiologists' mental and physical health and improve their productivity. For instance, AI-assisted imaging data interpretation and structural emergency radiology report generation could dramatically streamline and improve the clinical workflow in emergency radiology.

## Grand Challenges in Cardiothoracic Imaging

Cardiothoracic imaging mainly deals with diagnosis of pulmonary, cardiac, and vascular diseases, and it is closely related to disciplines including pulmonary medicine, thoracic surgery, cardiology, vascular surgery, oncology, and pathology. One of the grand challenges in cardiothoracic imaging concerns with how to effectively deal with the anatomic, mechanical, physiologic, physiopathological and therapeutic cardiopulmonary correlations (5), thus advancing the sub-field of integrated cardiothoracic imaging. Second, developing faster and smarter cardiothoracic imaging data acquisition and reconstruction technologies remains as a grand challenge, partly due to the cardiac and pulmonary motion. Those novel technologies that can effectively compensate or correct cardiac/pulmonary motion will be much needed. Third, from a computational perspective, increasing effort has been put into the integration of novel AI technologies into cardiothoracic imaging data interpretation and understanding, e.g., computational simulation and modeling of cardiac motion patterns.

## Grand Challenges in AI in Radiology and Future Directions

A growing number of academic and industrial efforts have been put in developing new AI technologies for radiology (6) and promising pilot results have been reported. However, grand challenges still exist to take the full advantages of AI in advancing radiology, and those challenges have been already discussed in the literature (7). From our perspectives, there are at least four urgent challenges that should be addressed by the field. First, new AI technologies should be developed for fast and high-quality imaging data reconstruction, with potentially smaller doses of intravenous contrast material and lower radiation dose in some scenarios. These new imaging data acquisition technologies will be very beneficial to patients, radiologists and radiology clinical flow. Second, as annotated imaging data is typically required for training AI models and algorithms, it is much needed to develop effective automated labeling and annotation methods to produce training data for radiology AI research. These automated annotation and labeling technologies will significantly improve current manual labeling/annotation processes that are both costly and time-consuming. Third, although AI is deemed to be promising in radiology, it requires large-scale, well-curated, well-integrated, and controlled dataset for training and testing AI algorithms. Thus, developing novel paradigms for effective, privacy-enabled, and safe radiology data sharing is urgently needed. Fourth, as AI systems are becoming more prominent in radiology practice, it is important to develop novel human-machine interaction frameworks for radiologists to smoothly interact and cooperate with AI systems. These novel AI-radiologists cooperation and teamwork models will both significantly improve radiologists' efficiency and reduce AI system's risks.

## Author Contributions

The author confirms being the sole contributor of this work and has approved it for publication.

## Conflict of Interest

The author declares that the research was conducted in the absence of any commercial or financial relationships that could be construed as a potential conflict of interest.
